# Fenestrated Thoracic Endovascular Repair for Acute Type B Aortic Dissection with Isolated Left Vertebral Artery: A Case Report

**DOI:** 10.3400/avd.cr.23-00067

**Published:** 2024-01-17

**Authors:** Yuta Yamada, Takao Ohki, Naoki Toya, Eisaku Ito, Hikaru Nakagawa

**Affiliations:** 1 Division of Vascular Surgery, Department of Surgery, The Jikei University Kashiwa Hospital, Kashiwa, Chiba, Japan; 2 Division of Vascular Surgery, Department of Surgery, The Jikei University School of Medicine, Tokyo, Japan

**Keywords:** vertebral artery, dissecting aneurysm, endovascular procedure

## Abstract

Thoracic endovascular aortic repair (TEVAR) of acute uncomplicated type B aortic dissection (uTBAD) has been discussed for its potential to prevent future aortic events. We present a fenestrated TEVAR in the case of an 86-year-old man with acute uTBAD with an isolated left vertebral artery (ILVA). The ILVA originated from the distal side of the left subclavian artery, the left subclavian artery, and the intramural hematoma with an ulcer-like projection extended close to the left subclavian artery. We selected a fenestrated stent graft to achieve a proximal healthy landing. This case demonstrates that a fenestrated stent graft for acute uTBAD is useful for preserving arch vessels.

## Introduction

Thoracic endovascular aortic repair (TEVAR) has recently been discussed in treating acute uncomplicated type B aortic dissection (uTBAD) if the aorta enlarges rapidly.^1^^,^^2)^ However, surgeons must consider the preservation of cervical branches when the dissection extends into the aortic arch. Here, we report the treatment of uTBAD with an isolated left vertebral artery (ILVA) arising directly from the aortic arch adjacent to the left subclavian artery (LSA) using a semi-custom-made fenestrated stent graft.

## Case Report

**Figure 1:**
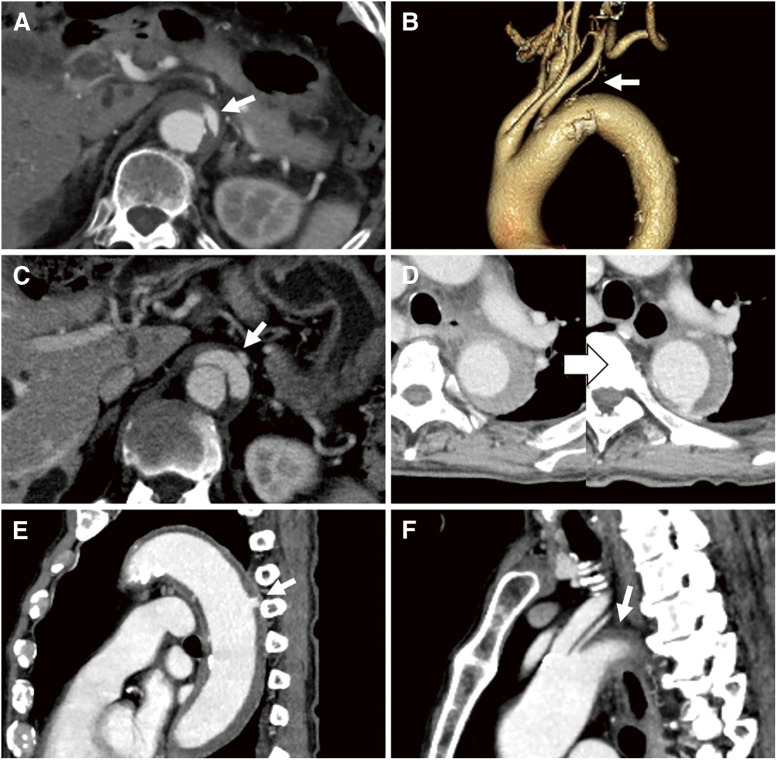
Fig. 1 Preoperative computed tomography. (**A**) Computed tomography on the day of admission reveals a tear in the aortic dissection at the origin of the celiac artery. (**B**) Computed tomography shows the shared origin of brachiocephalic and left common carotid arteries. The left vertebral artery arising directly from the aortic arch on the distal side of the left subclavian artery is indicated with an arrow. (**C**) Computed tomography performed on the 11th day post-admission indicates dilation of the false lumen. (**D**) Computed tomography performed on the day of admission and the 11th day post-admission (left to right) indicates enlargement of the descending aorta from 43 to 48 mm. (**E**) Computed tomography performed on the 11th day post-admission reveals an ulcer-like projection in the descending aorta. (**F**) Computed tomography performed on the 11th day post-admission reveals that the intramural hematoma with ulcer-like projection extended close to the left subclavian artery and isolated left vertebral artery.

**Figure 2:**
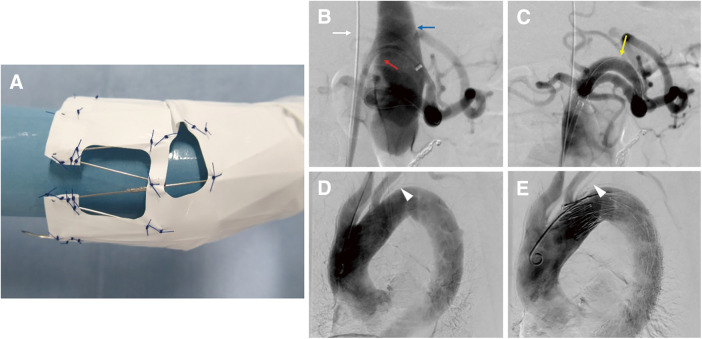
Fig. 2 Angiography during endovascular repair on the 31st day after aortic dissection onset. (**A**) Najuta stent graft with two fenestrations is shown. Fenestrations were designed to preserve blood flow through the left subclavian and isolated left vertebral arteries. The custom-made device required a few weeks to create. Note the fact that the graft material is largely unattached to the stent thereby allowing for active seal. (**B**) An angiographic image shows the true lumen (white arrow), false lumen (blue arrow) and the orifice of the celiac artery tear (red arrow). (**C**) Angiography after implantation of the Viabahn-covered stent (yellow arrow) reveals the absence of blood flow in the false lumen and the preservation of the celiac artery circulation. (**D**) Angiography indicates an isolated left vertebral artery (arrowhead) arising directly from the aortic arch just distal to the left subclavian artery. (**E**) Angiography after implantation of Najuta stent graft indicates the preservation of the isolated left vertebral artery (arrowhead) blood flow is shown.

**Figure 3:**
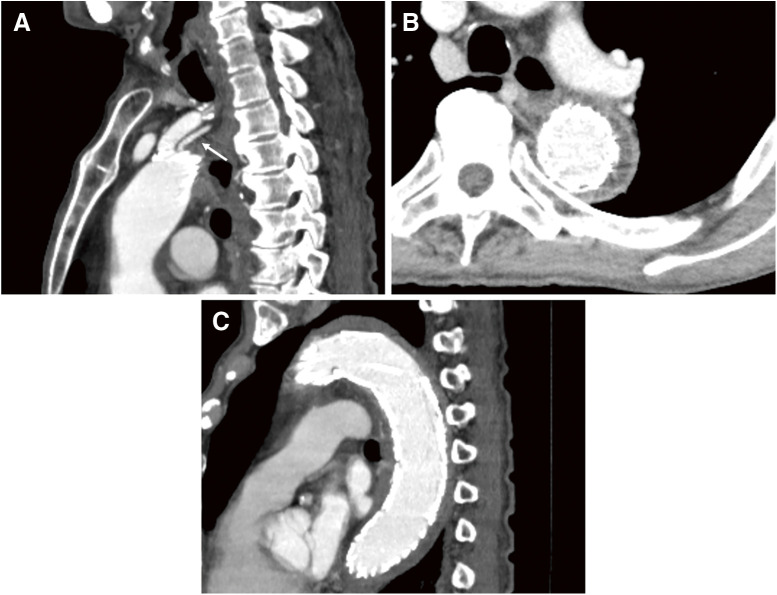
Fig. 3 Postoperative computed tomography. A postoperative computed tomography shows preserved isolated left vertebral artery blood flow (arrow, **A**); good aortic remodeling and descending aorta diameter reduction to 40 mm (**B**); and the absence of an ulcer-like projection in the descending aorta (**C**).

An 86-year-old man presented to our hospital with a 2-day history of chest and back pain. Contrast-enhanced computed tomography (CT) revealed that he had suffered a ruptured pseudoaneurysm due to segmental arterial mediolysis of the second jejunal branch of the superior mesenteric artery, which was treated successfully using transcatheter arterial embolization. CT also showed type B aortic dissection from zone 3 to zone 6 (TBAD 3,6), an ulcer-like projection (ULP) of the mid-descending aorta, and a tear in the dissection at the orifice of the celiac artery (CA) (**Fig. 1A**). In addition, an aberrant arch anatomy was noted, comprising a bovine arch and left vertebral artery directly arising from the aortic arch on the distal side of the LSA (**Fig. 1B**). The patient experienced pain two days prior to admission, which was presumed to be the date of onset of aortic dissection. A CT scan performed 11 days after admission showed expansion of the patent false lumen at the CA (**Fig**. **1C**), enlargement of the descending aorta from 43 to 48 mm (**Fig. 1D**), and ULP in the descending aorta (**Fig. 1E**). Pre-emptive TEVAR was planned for the enlargement; however, it was unknown whether the origin of the dissection was the ULP or the CA tear. Therefore, we decided to perform entry closure of the ULP and CA tear with TEVAR and a bridging covered stent, respectively. For TEVAR, a Najuta fenestrated semi-custom-made stent graft (SB Kawasumi Laboratories, Inc., Kanagawa, Japan) was selected to preserve the LSA and ILVA blood flow against the aberrant arch anatomy (**Fig. 2A**). As the intramural hematoma with ULP extended close to the LSA (**Fig. 1F**), a Najuta stent graft seemed necessary for a proximal healthy landing and additional sealing zone. The Najuta stent graft was designed for his arch (outer diameter: 31 mm, length: 200 mm), and waiting for its preparation, the operation was performed on the 31st day of admission. Under general anesthesia, the accesses were obtained by bilateral common femoral artery puncture and the right brachial artery via surgical cut-down. A Viabahn covered stent (W. L. Gore & Associates, Inc., Flagstaff, AZ, USA) was placed from the aortic true lumen to the detached orifice of the CA via the false lumen to close the distal tear. An angiography image after implantation showed that the Viabahn stent was bridging the CA, and the contrast leakage into the false lumen was almost lost (**Figs. 2B** and **2C**). Conformable Gore TAG (CTAG; W. L. Gore & Associates, Inc.) was placed in the descending aorta. A guidewire was then advanced from the right brachial artery to the right femoral artery. Then, the Najuta stent graft was placed in zone 2, however it migrated distally, and LSA and ILVA blood flow were preserved by only proximal fenestration. Completion angiography showed no entry flow and blood flow preservation in the LSA and ILVA (**Figs. 2D** and **2E**). A CT scan performed two weeks after the operation revealed that the ILVA was preserved, and the descending aortic diameter decreased from 48 mm to 40 mm (**Fig. 3**). The patient’s postoperative course was uneventful.

## Discussion

ILVA is a type of aortic arch anomaly with a reported incidence of 0.8%–5.4% globally.^3^^–^^5)^ Here, the patient had an anomaly of Adachi and Williams’ classification type BE,^3^^,^^4)^ an extremely rarely reported form of ILVA combined with a bovine arch. Generally, debranching bypass, branched stent graft, the chimney technique, and retrograde in situ branched stent are effective methods for preserving cervical branches, as demonstrated by several studies.^6^^,^^7)^ These techniques have been used successfully in the treatment of patients with Adachi and Williams type C aortic arch anomalies, which are defined as cases in which the left vertebral artery arises from the proximal side of the left LSA. In some cases, ILVA reconstruction via transposition and the chimney technique has been used.^3^^,^^4^^,^^8^^,^^9)^ However, in the present case, surgical exposure of the ILVA was expected to be difficult and highly invasive, and endovascular treatment was selected as the preferred approach. Herein, we report a case in which the ILVA was preserved using a custom-made fenestrated stent graft. The Najuta stent graft has a structure in which an ePTFE graft is located outside the Z-stent and is largely unattached to the stent (**Fig. 3**). This feature allows the graft to expand with blood flow, thereby functioning like a windsock and creating an active seal. For this reason, the radial force of the Najuta is minimized and, therefore, may contribute to the prevention of new entries proximally and distally.^10)^ It is regrettable that the stent graft migrated distally in this case. Due to its design, Najuta directly receives cardiac output during deployment, requiring careful attention. It has been reported that the administration of drugs, such as adenosine, and the use of rapid pacing during deployment can effectively aid in the precise positioning of the stent graft by inducing hypotension in the patient.^11^^,^^12)^ We should consider employing these methods. Furthermore, in the present case, bridging covered stent placement was effective for large tears in the CA.

## Conclusion

We performed fenestrated TEVAR with ILVA preservation using the Najuta stent graft to treat uTBAD in a patient with the extremely rare Adachi and Williams aortic arch type BE. This case indicates that fenestrated TEVAR is a useful option for preserving the cervical branches, including the ILVA.

## Informed Consent

The patient provided informed consent for case details and imaging studies to be reported.

## Author Contributions

Study conception: YY, NT, EI, and HN

Data collection: YY, NT, EI, and HN

Analysis: YY, NT, EI, and HN

Investigation: YY, NT, EI, and HN

Manuscript preparation: YY

Funding acquisition: none

Critical review and revision: all authors

Final approval of the article: all authors

Accountability for all aspects of the work: all authors.

## Disclosure Statement

The authors do not have any conflicts of interest to declare.
